# Phase Synchronization Dynamics of Neural Network during Seizures

**DOI:** 10.1155/2018/1354915

**Published:** 2018-10-15

**Authors:** Hao Liu, Puming Zhang

**Affiliations:** School of Biomedical Engineering, Shanghai Jiao Tong University, Shanghai 200240, China

## Abstract

Epilepsy has been considered as a network-level disorder characterized by recurrent seizures, which result from network reorganization with evolution of synchronization. In this study, the brain networks were established by calculating phase synchronization based on electrocorticogram (ECoG) signals from eleven refractory epilepsy patients. Results showed that there was a significant increase of synchronization prior to seizure termination and no significant difference of the transitions of network states among the preseizure, seizure, and postseizure periods. Those results indicated that synchronization might participate in termination of seizures, and the network states transitions might not dominate the seizure evolution.

## 1. Introduction

Epilepsy is a common chronic neurological disease characterized by recurrent epileptic seizures which may cause severe physiological and psychological damage to patients [1]. Tracking the evolution of seizure may be helpful to understand the generation, propagation, and termination mechanism of seizure and to improve the therapy of epilepsy.

Since epilepsy is increasingly considered as a network-level disorder, more and more attention has been paid to dynamic brain network analysis, which has provided new perspectives and insights into the nature of epilepsy [2, 3]. Meanwhile, evidence has shown that seizures result from dynamics of network organization characterized by dynamical evolution of synchronization [4, 5]. Traditional ideas hold that the hypersynchronous activity is the hallmark of epileptiform activity [6]. Recently, a high level of synchronization has been observed prior to the termination of seizures in several studies, which may provide helpful clues to understand the mechanism of seizure termination. Schindler et al. explored status epilepticus electroencephalogram (EEG) signals based on the eigenvalue spectrum of the equal-time correlation matrix, in which EEG signals were recorded by either intracranial or surface electrodes from patients, and found that synchronization fluctuated at relatively low levels during ongoing epileptiform activity and increased before seizures termination [7]. Then, they established a functional network by computing a cross-correlation matrix based on EEG signals recorded via implanted strips, grids, or depth electrodes from patients undergoing presurgical evaluation for drug-resistant epilepsy and found a global increase of synchronization prior to seizure termination [8]. Furthermore, Evangelista et al. used the nonlinear correlation coefficient to study functional connectivity between the thalamus and selected cortical regions based on stereo-electroencephalogram (SEEG) recorded from patients with drug-resistant mesial temporal lobe epilepsy and found that, at the end of seizure, the global synchronization index and the thalamic synchronization index negatively correlated with seizure duration, which indicated that the thalamocortical synchronization contributed to seizure termination [9]. In addition, Zhang et al. studied on amygdala-kindled seizures in mice and showed that the synchronization measured by mutual information between the thalamus and hippocampus increased prior to the epileptiform discharges' termination [10]. However, by computing Gabor atom density of individual electrode signals from patients with complex partial seizures of both mesial temporal or neocortical onset, Afra et al. found that synchronous seizure termination was a common pattern, which meant most of the ictal activities terminated in all the recording electrodes simultaneously or within 5 s between each other, whereas an asynchronous termination pattern also existed [11]. So, synchronization changes prior to the termination of seizures are debatable and to be further explored.

Several methods, such as coherency [12, 13], mutual information [10, 14], partial-directed coherence [15–17], directed transfer function [18, 19], and phase synchronization [20, 21], have been used to measure the interactions among different areas of the brain to establish a network. Among these methods, phase synchronization measures the interactions between rhythmic signals by detecting the instantaneous phase and is unaffected by the amplitude of signals, which is a useful method to study the synchronization between brain areas, especially when the relationship is too weak to be detected by other measures [14, 22]. Because of the nonlinear property of epileptic brain activities, phase synchronization, as a nonlinear method, has been applied to measure the synchronization between different brain areas [6, 23]. In this study, brain networks would be established by computing phase synchronization between different brain areas.

Studies have applied different indices to characterize brain networks and investigate synchronization dynamics, such as eigenvalue spectrum of the correlation matrix [7], eigenvalue ratio of the Laplacian matrix of the cross-correlation matrix [8], and so on. Evangelista et al. calculated the average of the nonlinear correlation coefficient of all pairs of signals in each time window in regions of interest as a global synchronization index, which was used to measure the thalamocortical synchrony based on SEEG signals [9]. Similarly, in this study, we would calculate the average degree of nodes in each network, which represented the mean phase synchronization of the network, and investigate the dynamics of the average degree prior to seizure termination to investigate the role of synchronization.

Similar network states denote similar brain connectivity patterns. Clustering of the networks over time windows were used to identify a set of distinct network clusters denoting different brain states [24, 25]. Burns et al. investigated the state-space dynamics of patients with partial epileptic seizures based on unsupervised clustering of eigenvector centrality vectors and characterized seizures by a set of network states [24]. Khambhati et al. defined a network state to be the set of all configuration vectors that exhibited a similar pattern of functional connectivity and tracked the dynamic network reconfiguration by clustering time windows (configuration vectors) with similar network geometry via community detection during the seizure generation, propagation, and termination [25]. They found that, during the preseizure epoch, the network demonstrated rapid reconfiguration, but during the seizure epoch, the network showed slower reconfiguration. Liu et al. explored the state transitions of networks of epileptiform discharges in hippocampal slices based on clustering of degree vectors and identified two network states during the ictal-like discharges which represented tonic and clonic phases, respectively [26]. Since the network states characterized by brain connection patterns represent different stages of the seizure, we would investigate the dynamics of network states during preseizure, seizure, and postseizure periods by using unsupervised clustering of degree vectors to explore the reconfiguration of networks.

The rest of the paper is organized as follows.  Section 2 presents the information of data sets and the algorithms for calculating phase synchronization and network states. The results of dynamics of phase synchronization and network states over preseizure, seizure, and postseizure periods are presented in Section 3.  Section 4 presents the conclusion of this study.

## 2. Materials and Methods

### 2.1. Patient Data Sets

We retrieved ECoG signals recorded from 11 refractory epilepsy patients (Mayo Clinic, Rochester, MN) undergoing implantation of subdural electrodes to localize the seizure onset zone via the International Epilepsy Electrophysiology Portal (IEEG Portal, http://www.ieeg.org), including complex partial seizure and complex partial with secondary generalization seizure. Detailed patient information is given in Table 1. Each of the data sets includes ECoG signals and annotations of seizure time. The ECoG signals were sampled at 500 Hz. We analyzed the largest grid of electrodes in each case to investigate the epileptic network of a local area. The grid sizes are listed in Table 1, which denote the total number and the arrangement of electrodes.

### 2.2. Data Analysis

#### 2.2.1. Data Preprocessing

We analyzed the preseizure, seizure, and postseizure periods of 22 seizures from 11 patients (2 seizures from each patient). A seizure period denoted the time between seizure onset and termination, and the corresponding preseizure period and postseizure period were defined as the same time intervals of the seizure period before the seizure onset and after the seizure termination, respectively. Since the ECoG signals had high signal-to-noise ratio, we only eliminated the bad channels seriously affected by noise and did not proceed other preprocessing to obtain broadband signals. Then, the theta (4–8 Hz), alpha (8–13 Hz), beta (13–30 Hz), and gamma (30–45 Hz) signals were extracted with finite impulse response filters provided by EEGLAB [27, 28] to ensure zero-phase distortion for further phase synchronization analysis.

#### 2.2.2. Phase Synchronization

The mean phase coherence was calculated to measure the phase synchronization between each pair of ECoG signals. Firstly, for one channel of ECoG signals *x*_1_(*t*), the Hilbert transform [29] was computed as follows:

(1) $$\begin{array}{c} \text{H}\left[x_{1}\left(t\right)\right]=\frac{1}{\pi }\text{P}.\text{V}.\int_{-\infty }^{\infty }\frac{x_{1}\left(\tau \right)}{t-\tau }\text{d}\tau , \end{array}$$

where P.V. means the Cauchy principal value. Then, the instantaneous phase *ϕ*_1_(*t*) of *x*_1_(*t*) was defined as

(2) $$\begin{array}{c} \phi_{1}\left(t\right)=\arctan\frac{\text{H}\left[x_{1}\left(t\right)\right]}{x_{1}\left(t\right)}, \end{array}$$

and the mean phase coherence λ between a pair of ECoG signals *x*_1_(*t*) and *x*_2_(*t*) was calculated as

(3) $$\begin{array}{c} \lambda =\frac{1}{N}\left|\overset{N}{\underset{t=1}{\sum }}{\text{e}}^{\text{j}\left[\phi_{1}\left(t\right)-\phi_{2}\left(t\right)\right]}\right|. \end{array}$$

where *ϕ*_1_(t) and *ϕ*_2_(t) were the instantaneous phases of the signals *x*_1_(*t*) and *x*_2_(*t*), respectively and *N* was the number of samples in each time window. λ ranges from 0 to 1, and λ=0 means no phase synchronization, whereas λ=1 indicates the perfect phase synchronization.

To qualify the dynamics of phase synchronization, 1-s moving windows were used with 50% overlap, and the mean phase coherence was calculated for each pair of signals and averaged over all samples in each window (here, the number of samples in each window is *N*=500). So, a *M* × *M* phase synchronization connectivity matrix (*M* is the number of electrodes in the grid) was obtained in each time window for each subject. In this way, we would obtain a set of phase synchronization connectivity matrices during preseizure, seizure, and postseizure periods denoting a series of brain networks.

#### 2.2.3. Network Analysis

The network was constructed by the connectivity matrix in each time window, in which the nodes referred to the ECoG channels and the edges were defined as the mean phase coherence value.

In this study, we used the degree to characterize the network. The degree of a node in a network was defined as the sum of the weights of edges which were connected with the given node [2], and the degree vector was formed of the degrees of all nodes in each time window. The mean phase synchronization of the network was computed as the average value of the degrees of all nodes and was used to measure the phase synchronization of the whole network.

To investigate the transitions of network states, the degree vectors along time were clustered into a set of states by using an agglomerative hierarchical method on Ward's criterion [30]. An agglomerative hierarchical clustering started with each object as a single cluster and then, merged the two nearest clusters repeatedly until only a single cluster remained, and the Ward's criterion was used to measure the proximity (distance) between two clusters through the increase of the sum of the squared error (SSE) resulting from merging the two clusters. The objective function SSE is defined as follows:

(4) $$\begin{array}{c} \text{SSE}=\overset{\text{K}}{\underset{i=1}{\sum }}\underset{x\in C_{i}}{\sum }\text{dist}{\left(c_{i},x\right)}^{2}, \end{array}$$

where K means the number of clusters, *C*_*i*_ is the cluster *i*, *x* means an object, *c*_*i*_ is the centroid of the cluster *i*, and the dist denotes the standard Euclidean distance. Then, the objective function would be minimized to merge the nearest clusters. The optimal number of clusters was determined by the largest second derivative in the distance curve of clustering [26]. Then, we obtained a set of network states and computed the rate of the state change by dividing the number of network state changes by corresponding length of time to investigate the reorganization of brain networks during the preseizure, seizure, and postseizure periods.

#### 2.2.4. Statistical Analysis

The Friedman test with the post hoc Dunn's multiple comparison test was performed to test the difference among multiple groups, with p < 0.05 indicating the significant difference.

## 3. Results and Discussion

### 3.1. Synchronization Increasing prior to Seizure Termination

**Figure 1:**
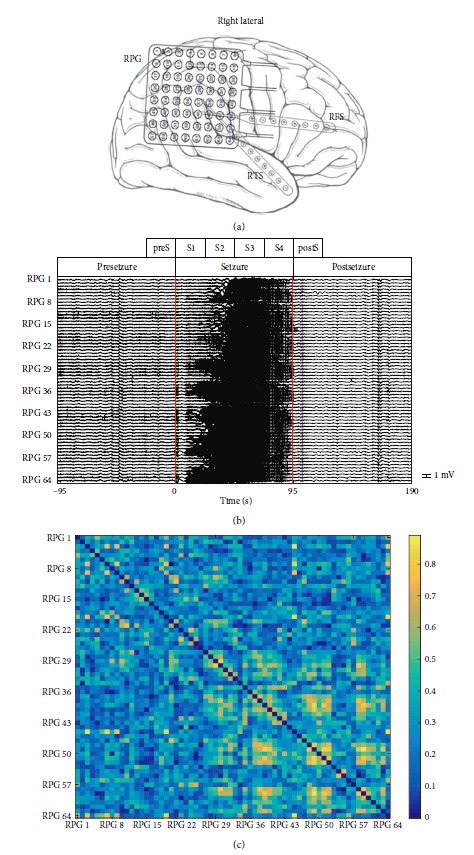
ECoG information of Study 037 and one example of the corresponding phase synchronization connectivity matrix. Electrodes positions of the grid RPG are shown in (a) and 64 channels of ECoG signals recorded from the grid are shown in (b). The left red vertical line means the onset time of this seizure and the right red vertical line represents the termination time. The seizure period is divided into four stages evenly, named “S1–S4”, and preS and postS represent the period before the onset and after the termination of seizure with a duration of a quarter of the seizure period, respectively. Phase synchronization connectivity matrix in the 47–48 s time window of broadband signals is displayed in (c).

For a seizure of patient Study 037 with an 8 × 8 grid, Figure 1(a) represented the grid and strips' location obtained from the IEEG portal, and the recording data from the grid named “RPG” were analyzed.  Figure 1(b) demonstrated 64 channels of ECoG signals recording from the grid RPG, where the left red vertical line denoted the onset time of this seizure and the right red vertical line denoted the termination time. This seizure lasted for 95 s, and we selected the same duration before the onset of seizure as preseizure period and the same duration after the termination of seizure as postseizure period. Then, the seizure period was divided into four periods evenly, named “S1–S4”, and preS and postS denoted the periods before the onset and after the termination of seizure with a duration of a quarter of seizure period, respectively.  Figure 1(c) shows the phase synchronization connectivity matrix in the 47-48 s time window of broadband signals.

**Figure 2:**
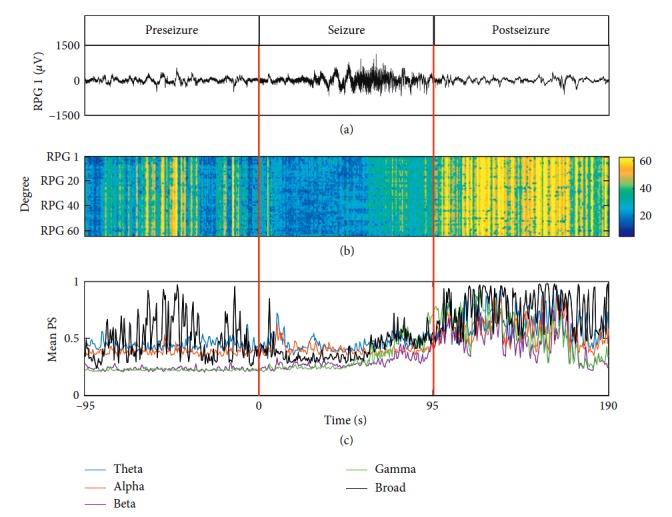
Degree vectors and corresponding mean phase synchronization of Study 037. The left red vertical line denotes the onset of this seizure, and the right red vertical line represents the termination of this seizure. (a) shows the #1 channel of the grid signals (RPG1) of the seizure of Study 037. The degree vectors of broadband signals along time are shown in (b), and mean phase synchronization (mean PS) of different frequency components are displayed in (c).

For this patient, the degree vectors, calculated based on broadband signals and formed of the degree of each node during the preseizure, seizure, and postseizure periods, are displayed in Figure 2. As shown in Figure 2, the mean phase synchronization of broadband signals was at a high level in the preseizure period and decreased in the early stage of the seizure period, then it increased significantly in the late stage of the seizure period and remained at the high level in the postseizure period. Moreover, the mean phase synchronization in different frequency bands of Study 037 was different, especially in preseizure periods, in which alpha and theta components showed higher mean phase synchronization than beta and gamma components. However, the mean phase synchronization in all frequency bands increased in the late stage of the seizure period and remained at the high level in the postseizure period.

**Figure 3:**
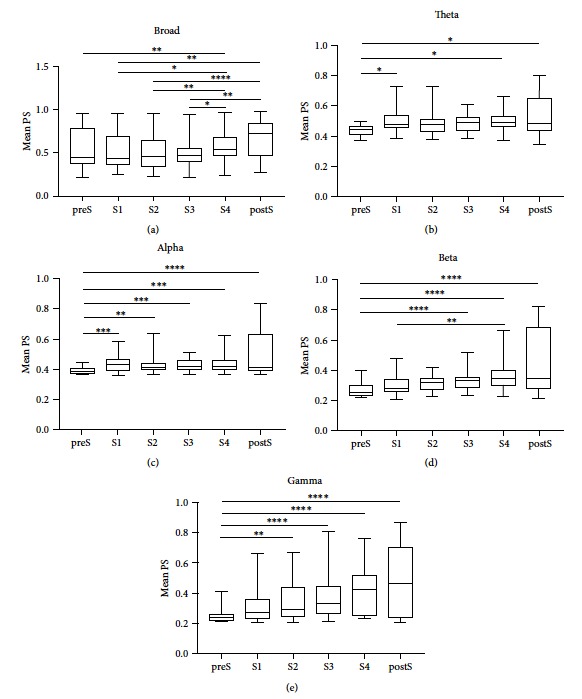
Statistical analysis results of mean phase synchronization of broadband signals and different frequency band signals (theta, alpha, beta, and gamma) are displayed in (a), (b), (c), (d), and (e), respectively. The middle line of the box corresponds to the median, and the 25% and 75% percentiles are the lower and upper borders of each box, respectively. The whiskers correspond to the total range of the data. (Friedman test with post hoc Dunn's multiple comparison test; *n*=22, ^*∗*^*p* < 0.05, ^*∗∗*^*p* < 0.01, ^*∗∗∗*^*p* < 0.001, and ^*∗∗∗∗*^*p* < 0.0001).

The statistical analysis results of mean phase synchronization in broadband and different frequency bands across 22 seizures from 11 patients are shown in Figure 3. As shown in Figure 3(a), the mean phase synchronization of broadband signals in S4 was significantly larger than those in preS (*p* < 0.01), S1 (*p* < 0.05), S2 (*p* < 0.01), and S3 (*p* < 0.05). Moreover, the mean phase synchronization of broadband signals in postS was significantly larger than those in S1 (*p* < 0.01), S2 (*p* < 0.0001), and S3 (*p* < 0.01). The mean phase synchronization in the theta band was displayed in Figure 3(b), and the mean phase synchronization in S1, S4, and postS was significantly larger than that in preS. In the alpha band, the mean phase synchronization in S1, S2, S3, S4, and postS was significantly higher than that in preS (Figure 3(c)). Moreover, the mean phase synchronization in S4 was significantly larger than that in S1, and those in S3, S4, and postS were higher than those in preS in the beta band (Figure 3(d)). As shown in Figure 3(e), the mean phase synchronization in S2, S3, S4, and postS was significantly higher than that in preS in the gamma band. There was no statistical difference between the other pairs. The results demonstrated that the mean synchronization of the network had a significant increase prior to seizure termination and remained at a high level after seizure termination, especially in alpha, beta, and gamma bands.

Several research studies explored synchronization based on broadband signals [7–9], and Li et al. explored the dynamics of phase synchronization of different frequency components and found that the phase synchronization increased before the seizure termination across almost all frequencies [21]. Our study also displayed similar results, which meant multiple brain areas with multiple frequency components may be involved in seizure termination.

Our results confirmed that the phase synchronization increased at the late stage of seizure compared with the early periods and remained high after seizure. In recent years, several studies have reported that a high level of synchronization was observed prior to the termination of seizures in vitro and in vivo models and human seizures [7–10, 21, 31].

The increasing synchronization of neural activities may be considered as an emergent self-regulatory mechanism of seizure termination [7]. Meanwhile, Sobayo and Mogul found that deep brain stimulation was more effective to terminate seizures when the frequency reflected the endogenous synchronization of naturally terminated seizures in a chronic rat epilepsy model [32], which supported that synchronization might cause seizure termination. However, Majumdar et al. discussed the cause and effect relationship between the seizure termination and the increase of synchronization, and they concluded that the synchronization was an effect [33]. So far, it is not conclusive that the synchronization is the cause or effect of the seizure termination. In addition, the degree of synchronization was measured based on phase synchronization in this study, which characterized different aspects of synchronization compared with other methods such as correlation and coherence. Further studies will be needed to explore the cause and effect relationship between the seizure termination and synchronization, and it should be more careful to apply synchronization to intervention of epilepsy especially.

### 3.2. No Significant Difference of Network State Change Rate during Periseizure

**Figure 4:**
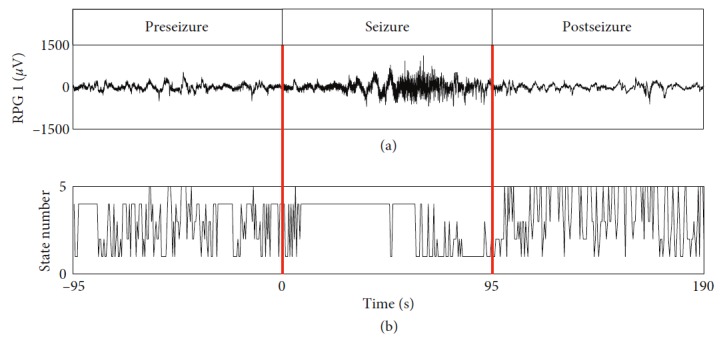
Transitions of network states of Study 037 during preseizure, seizure, and postseizure periods. The left red vertical line denotes the onset of this seizure, and the right red vertical line represents the termination of this seizure. (a) shows the #1 channel of the grid signals (RPG1) of the seizure of Study 037. The network states resulted from clustering of degree vectors are displayed in (b), in which each state number denotes a network state.

To investigate the transitions of network states, a set of network states were obtained by applying agglomerative hierarchical clustering to the set of degree vectors, which represented different brain connectivity patterns during preseizure, seizure, and postseizure periods. For example, the transitions of network states based on broadband signals during periseizure of Study 037 are shown in Figure 4, in which each state number denotes a network state. The network states switched among state 1, state 2, state 3, state 4, and state 5 frequently during the preseizure and postseizure periods. However, the frequency of network state changes decreased significantly during the seizure period, and the state 4 dominated the most of seizure periods.

**Figure 5:**
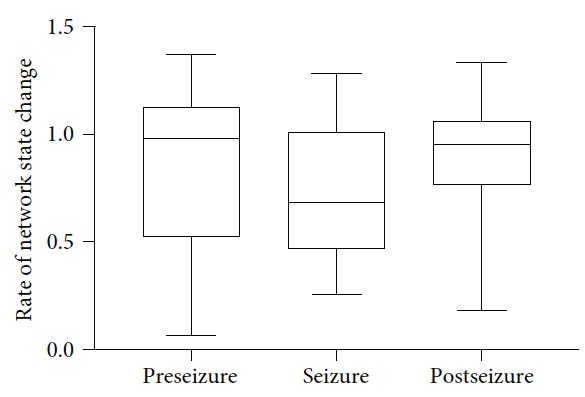
Statistical analysis results of network state change rate. The middle line of the box corresponds to the median, and the 25% and 75% percentiles are the lower and upper borders of each box, respectively. No significant difference exists among those pairs. (Friedman test with post hoc Dunn's multiple comparison test; *n*=22).

The statistical analysis results across 22 seizures from 11 patients are shown in Figure 5. Although there is a downward trend of the network state change rate from the preseizure period to the seizure period and an upward trend of that from the seizure period to the postseizure period, no significant difference exists among the three periods.

Previous studies have demonstrated that brain network transitions existed in seizure periods based on measuring the coherence between each pair of ECoG signals [24], and the network reorganization was faster during preseizure periods than seizure periods by calculating correlation of the pairs of signals [25]. By computing time-variant partial-directed coherence, Liu et al. found there were two network states during the ictal-like discharges in hippocampal slices and those two states represented tonic and clonic phases, respectively [26].

 Here, based on the phase synchronization, we also found the network transitions existed during the preseizure, seizure, and postseizure periods, and network state changes in the seizure period had a tendency to be less frequent compared with preseizure and postseizure periods. However, our statistical results did not show significant difference of the network state change rate among those periods, which might be due to that the networks were established by calculating the phase synchronization. Compared with those studies mentioned above, phase synchronization focused on measuring the difference of the instantaneous phase of rhythmic signals. So, our results were restricted to the network state changes based on phase synchronization, which did not dominate in evolution of seizure. Further research studies will be performed to track synchronization dynamics based on other methods such as partial-directed coherence and directed transfer function, which characterize the information flow direction and may be helpful to measure the interactions among signals.

## 4. Conclusions

In this study, phase synchronization was applied to measure the relationship between each pair of ECoG signals to establish the brain network, and the synchronization dynamics and network states evolution were tracked during preseizure, seizure, and postseizure periods. The results showed that synchronization increased prior to seizure termination and remained at the high level after seizure termination. Furthermore, there was no significant difference of the network states transition rate among the preseizure, seizure, and postseizure periods. Those results indicated that the phase synchronization might promote the seizure termination, and the role of network state transitions might not dominate the seizure evolution, which led to a deeper understanding of evolution of seizure. From a therapeutic perspective, these results might be helpful to explore new therapeutic methods focused on phase synchronization to terminate seizures.

## Figures and Tables

**Table 1:** Patient information.

Patient (IEEG portal)	Sex	Age (onset/surgery)	Seizure type	Grid size
Study 006	M	22/25	CP	6 × 8
Study 010	F	00/13	CP	6 × 8
Study 011	F	10/34	CP	6 × 8
Study 014	F	09/33	CP	8 × 8
Study 016	F	05/36	CPG	4 × 6
Study 020	M	05/10	CPG	4 × 6
Study 021	M	Unknown	CPG	6 × 8
Study 022	F	Unknown	CPG	4 × 6
Study 023	M	01/16	CP	8 × 8
Study 026	M	09/09	CP	8 × 8
Study 037	F	Unknown	CP	8 × 8

CP, complex partial; CPG, complex partial with secondary generalization. M, male; F, female.

## Data Availability

All ECoG data used to support the findings of this study are available at the International Epilepsy Electrophysiology Portal (IEEG Portal, http://www.ieeg.org). Researchers can access the data once they sign up this portal.
